# Updated science-wide author databases of standardized citation indicators

**DOI:** 10.1371/journal.pbio.3000918

**Published:** 2020-10-16

**Authors:** John P. A. Ioannidis, Kevin W. Boyack, Jeroen Baas

**Affiliations:** 1 Department of Medicine, Stanford University, Stanford, California, United States of America; 2 Department of Epidemiology and Population Health, Stanford University, Stanford, California, United States of America; 3 Department of Biomedical Data Science, Stanford University, Stanford, California, United States of America; 4 Meta-Research Innovation Center at Stanford (METRICS), Stanford University, Stanford, California, United States of America; 5 SciTech Strategies, Inc., Albuquerque, New Mexico, United States of America; 6 Research Intelligence, Elsevier B.V., Amsterdam, the Netherlands

## Abstract

This Formal Comment presents an update to citation databases of top-cited scientists across all scientific fields, including more granular information on diverse indicators.

There was great interest in the databases of standardized citation metrics across all scientists and scientific disciplines [1], and many scientists urged us to provide updates of the databases. Accordingly, we have provided updated analyses that use citations from Scopus with data freeze as of May 6, 2020, assessing scientists for career-long citation impact up until the end of 2019 (Table-S6-career-2019) and for citation impact during the single calendar year 2019 (Table-S7-singleyr-2019). Updated databases and code are freely available in Mendeley (https://dx.doi.org/10.17632/btchxktzyw). The original database (version 1) can also be found in https://data.mendeley.com/datasets/btchxktzyw/1, the updated (version 2) can also be found in https://data.mendeley.com/datasets/btchxktzyw/2, and any subsequent updates that might appear in the future will be generally accessible in https://dx.doi.org/10.17632/btchxktzyw.

S6 and S7 tabulated data include all scientists who are among the top 100,000 across all fields according to the composite citation index [2] when self-citations are included and/or when self-citations are not included. Furthermore, in the current update, Tables S6 and S7 include also scientists who are not in the top 100,000 according to the composite index but are nevertheless within the top 2% of scientists of their main subfield discipline, across those that have published at least five papers. Another new feature in this update is that Tables S6 and S7 include new columns showing for each scientist the rank of their composite citation index within their subfield discipline (with and without self-citations) and the total number of authors within the subfield discipline. For example, for Kevin W. Boyack, rank is 50 and 52 for the composite citation index with and without self-citations, respectively, among the total of 10,391 scientists whose main subfield discipline is “Information and Library Sciences.” This extension allows the inclusion of more comprehensive samples of top-cited scientists for fields that have low citation densities and therefore would be less likely to be found in the top 100,000 when all scientific fields are examined together. Comparisons of citation metrics are more meaningful when done within the same subdiscipline. Of course, even within the same subdiscipline, different areas may still possess different citation densities, and assessing citation indicators always require caution.

Field and subfield discipline categories use the Science-Metrix classification as in our previous work [1], but multidisciplinary journals that were previously not assigned to a Science-Metrix field or subfield [3] have now been assigned to a specific field and subfield using a character-based convolutional deep neural network. This machine learning approach was trained with a set consisting of over a million entries was found to be outperforming other approaches such as Wikipedia and Yahoo! Answers [4]. This allows a more accurate classification of scientists who publish many papers in multidisciplinary journals.

Tables S8 and S9 provide the 25th, 50th, 75th, 90th, 95th, and 99th percentile thresholds for each field and each subfield for career-long and single year 2019 impact based on citations and, separately, based on the composite indicator. The formula to calculate the composite indicator for career-long impact is derived by summing the ratio of log of 1 + the indicator value over the maximum of those indicator logs for 6 indicators (NC, H, Hm, NCS, NCSF, NCSFL) [3]:

$$c_{i}=\frac{\log{(NC}_{i}+1)}{\max\;\log\left(NC+1\right)}+\frac{\log{(H}_{i}+1)}{\max\;\log\left(H+1\right)}+\frac{\log\left({Hm}_{i}+1\right)}{\max\;\log\left(Hm+1\right)}+\frac{\log{(NCS}_{i}+1)}{\max\;\log\left(NCS+1\right)}+\frac{\log{(NCSF}_{i}+1)}{\max\;\log\left(NCSF+1\right)}+\frac{\log{(NCSFL}_{i}+1)}{\max\;\log\left(NCSFL+1\right)}$$

The formula to calculate the composite indicator for single year 2019 impact follows the same principle and only uses citations from publications published in 2019. Maximum log values across the population are in separate tables for career (S10) and single year 2019 (S11).

Given the increasing attention given to the analysis of self-citations, we also include in Tables S8 and S9 data for each discipline and each subdiscipline of the 95th and 99th percentile threshold for the percentage of self-citations and for the ratio of citations over citing papers within the set of selected top-cited researchers. Very high proportion of self-citations and/or ratio of citations over citing papers may or may not be justifiable and may require a closer look at the citation practices of these scientists. A percentage (4.9%) of the scientists who are in the top 2% of their subdiscipline for career-long impact when self-citations are included are no longer in the top 2% of their subdiscipline when self-citations are excluded, and 0.01% (*n* = 15) of these fall below the top 10%. Some scientists have extremely high ratios of citations over citing papers, far exceeding the 99th percentile threshold. Many papers by the same scientist may be fully legitimately often cited together in the same article. However, some authors have been found to manipulate peer-review to add multiple citations to their works [5,6].

Publications in author profiles currently have 98.1% average precision and 94.4% average recall [7]. Comments for correction of author profiles should be addressed to Scopus, preferably by use of the Scopus to ORCID feedback wizard (https://orcid.scopusfeedback.com/).
